# Ventricular activation and repolarization in response to physiological and conventional pacing using ultra-high-frequency electrocardiography

**DOI:** 10.1371/journal.pone.0344111

**Published:** 2026-04-13

**Authors:** Saúl Palacios, Radovan Smisek, Karol Curila, Uyen Nguyen, Frits W. Prinzen, Josef Halamek, Filip Plesinger, Pavel Jurak, Javier Ramos, Juan Pablo Martínez, Esther Pueyo

**Affiliations:** 1 BSICoS Group, Aragon Institute of Engineering Research, IIS Aragon, University of Zaragoza, Zaragoza, Spain; 2 Institute of Scientific Instruments, the Czech Academy of Sciences, Brno, Czech Republic; 3 Charles University and University Hospital Kralovske Vinohrad, Cardiocenter, 3rd Faculty of Medicine, Prague, Czech Republic; 4 Maastricht University Medical Centre (MUMC), Maastricht, The Netherlands; 5 Arrhythmias Unit, Department of Cardiology, Hospital Clinico Lozano Blesa, Zaragoza, Spain; 6 CIBER en Bioingeniería, Biomateriales y Nanomedicina, Zaragoza, Spain; University of Minnesota, UNITED STATES OF AMERICA

## Abstract

**Background:**

Physiological pacing targeting the cardiac conduction system is increasingly being adopted as an alternative to conventional right ventricular (RV) pacing for the treatment of bradyarrhythmias, although its effects on ventricular repolarization remain underexplored.

**Objective:**

This study evaluates depolarization and repolarization responses to different pacing techniques using ultra-high-frequency electrocardiograms (UHF-ECGs).

**Methods:**

Temporary pacing was performed at different cardiac areas in 178 patients with bradycardia. Depolarization was assessed via QRS duration (QRS_*d*_), QRS area (QRS_*a*_), ventricular dyssynchrony (e-DYS), and activation time dispersion (dAT computed from leads V1-V6 and dAT_4-6_ from leads V1-V6). Repolarization was analyzed using the corrected QT interval (QTc), T-wave area (T_*a*_), Periodic Repolarization Dynamics (PRD), and repolarization time dispersion (dRTc and dRTc_4-6_).

**Results:**

His bundle pacing (HBP) preserved ventricular activation patterns similar to spontaneous rhythm. Left bundle branch pacing (LBBP) induced moderate depolarization changes, primarily due to delayed right ventricular activation, while maintaining left ventricular synchrony. In particular, dAT showed no significant differences between HBP and spontaneous rhythm, while differences between LBBAP and spontaneous rhythm were significant but below 7 ms in median. When restricted to the left ventricle (LV), no significant differences in dAT_4-6_ were found between LBBAP and spontaneous rhythm. Importantly, e-DYS for HBP showed similar values to spontaneous rhythm, LBBP led to a significant reduction (median differences approximately 20 ms), and RVP was associated with a significant increase (above 15 ms in median). In line with these results, QRS_*d*_ and QRS_*a*_ showed the largest values for RVP. In terms of ventricular repolarization, median differences in the QTc interval between pacing modes and spontaneous rhythm were below 3 ms for HBP, above 1 ms for LBBP, and above 20 ms for RVP. All pacing modes led to a reduction in PRD, with the most marked reductions observed for LBBP, particularly for selective LBBP, with median changes with respect to spontaneous rhythm of 4.6 degrees. RT and RTc showed similar trends for all pacing techniques. T_*a*_, however, showed median differences with respect to spontaneous rhythm above 100 and 34 $\begin{array}{c} \mu \end{array}$ Vs when pacing the RV at the apex and the septum, respectively, whereas such median differences were below 16 μ Vs for HBP and below 2 μ Vs for LBBP.

**Conclusion:**

Physiological stimulation via HBP or LBBAP generates ventricular depolarization and repolarization responses that more closely resembles that of spontaneous rhythm, in high contrast to the largely different response induced by RV pacing. HBP and LBBAP have distinct technical characteristics, including differences in capture thresholds, lead stability, and procedural aspects. These techniques serve as alternatives to conventional RV pacing.

## 1 Introduction

Cardiac arrhythmias affect 1.5% to 5% of the general population and account for up to 20% of all deaths worldwide [1,2]. In patients with bradyarrhythmias who have very slow heart rates, pacemakers apply electrical stimulation to restore the heart’s electrical activity to a normal cardiac rhythm. For several decades, the main pacing technique for treating bradyarrhythmias has been right ventricular pacing (RVP). Common locations for RVP stimulation are the right ventricular apex (so-called right ventricular apex pacing, RVAP) and the septum (so-called right ventricular septal pacing, RVSP). Despite the successful results of RVP for the treatment of bradyarrhythmias, numerous studies have shown its detrimental effects in terms of electrical and mechanical dyssynchrony of the ventricles, which predispose patients to develop mitral and tricuspid regurgitation, atrial fibrillation, cardiomyopathy, heart failure, and death [2–5].

As an alternative to the conventional, nonphysiological RVP, physiological pacing techniques have been proposed that stimulate the specialized cardiac conduction system to provide more physiological ventricular activation [6]. These techniques include His-bundle pacing (HBP) and left bundle branch (LBB) area pacing (LBBAP). HBP in patients who required pacemaker implantation was initially described by Deshmuk et al. [7] and, since then, its feasibility and safety have been demonstrated in different investigations. Importantly, HBP has been associated with a significant reduction compared to RVP in the combined endpoint of all-cause mortality, hospitalization for heart failure, and upgrade to biventricular pacing [8]. Recent studies indicate that, when implemented using a standardized and meticulous implantation technique, HBP can provide stable pacing parameters and favorable electrical outcomes [9,10]. Nevertheless, certain technical and procedural considerations have been reported in clinical practice, such as the anatomical complexity of the His bundle, its small size and surrounding fibrous tissue, as well as the variability of pacing capture thresholds and the potential for bundle injury during lead implantation [11–15]. LBBAP, by delivering pacing distal to the His bundle to achieve either left bundle branch capture or left ventricular septal myocardial pacing, has been described as facilitating lower and more consistent capture thresholds, while producing narrow paced QRS complexes and a high degree of left ventricular (LV) synchrony, particularly in patients with LBB block (LBBB) [11,14,15]. Despite the growing evidence supporting both HBP and LBBAP, larger randomized studies are still warranted to further assess feasibility, long-term safety, and clinical effectiveness [16], including direct comparisons of their respective effects on ventricular depolarization and repolarization.

To improve the characterization of ventricular activation in response to physiological pacing compared to conventional pacing, a number of studies have been conducted in recent years based on the standard electrocardiogram (ECG) and the vectorcardiogram (VCG). Most of these studies have evaluated the QRS morphology, duration, and area [17,18]. Additionally, ventricular depolarization patterns have been characterized from ultra-high-frequency ECG (UHF-ECG) recordings [19,20]. By proposing and validating novel measures of local depolarization duration and ventricular electrical dyssynchrony derived from UHF-ECG recordings, these studies have compared interventricular synchrony and the time for LV wall depolarization between various conventional and physiological pacing techniques [19,21–26].

Research on the effects of pacing on ventricular repolarization is much more limited than on ventricular activation. Some studies have quantified the duration of classical repolarization intervals, such as the heart rate-corrected QT interval, the JT interval, and the T-peak-to-T-end interval. These studies have shown a prolongation of the repolarization intervals following RVP but not following HBP or LBBAP [22,26–28]. Other studies have reported changes in the T wave, even if mostly from a qualitative point of view, without providing quantitative measures of T wave features [29–31].

Here, we extend the analysis of ECG ventricular depolarization and repolarization by processing UHF-ECG recordings from patients with physiological ventricular activation undergoing pacemaker implantation for bradycardia therapy. Both spatial and temporal features of the depolarization and repolarization phases of UHF-ECG recordings are quantified during spontaneous rhythm and in response to different types of conventional and physiological pacing. We hypothesize that physiological pacing techniques may lead to QRS and T wave patterns that better resemble those of spontaneous rhythm in patients without ventricular conduction abnormalities and that LBBAP techniques may be an equally effective option as HBP in reducing ventricular activation dyssynchrony, particularly in the LV, and in preserving ventricular repolarization characteristics.

## 2 Materials and methods

### 2.1 Study population

The study included mostly patients without bundle branch block and with an indication for pacemaker therapy due to bradycardia. Only 3.4% of the patients presented with left bundle branch block (LBBB), and 3.4% of them presented with right bundle branch block (RBBB). Six hundred and fifty-eight 14-lead UHF-ECG recordings, sampled at 5,000 Hz, were obtained from 178 patients (76 ± 7 years old, 68.5% male) using VDI UHF-ECG, VDI Technologies, Brno, Czech Republic, Cardiocenter of Faculty Hospital Kralovske Vinohrady, and the Third Medical Faculty of Charles University, Prague, Czech Republic. Almost 53% of the patients had atrioventricular block, and more than 38% had sick sinus syndrome. The remaining 9% of patients had other pacing indications, mainly bifascicular or trifascicular block and atrial fibrillation with planned AV junctional ablation. Other clinical characteristics of the study population are presented in Table 1. This research was conducted in accordance with the Helsinki Declaration as revised in 2016, was approved by the Ethics Committee of the Faculty Hospital Kralovske Vinohrady in Prague, and all subjects signed informed consent before enrollment.

**Table 1:** Baseline characteristics of the study population. Atrioventricular block (AV block), sick sinus syndrome (SSS), left bundle branch block (LBBB), right bundle branch block (RBBB), intraventricular conduction delay (IVCD).

**N = 178**	
Age (years), mean±SD	76 ± 7
Male, n (%)	122 (68.5)
**Comorbidities**	
Hypertension, n (%)	138 (77.5)
Diabetes, n (%)	58 (32.6)
Coronary artery disease, n (%)	61 (34.3)
Heart failure, n (%)	15 (8.4)
LV ejection fraction (%), mean±SD	58 ± 5
**Pacing indications**	
AV block, n (%)	94 (52.8)
SSS, n (%)	68 (38.2)
Other, n (%)	16 (9.0)
**QRS morphology**	
Narrow QRS, n (%)	151 (84.8)
LBBB, n (%)	6 (3.4)
RBBB, n (%)	6 (3.4)
IVCD, n (%)	15 (8.4)

The UHF-ECG recordings included 12 standard leads, as well as V7 and V8 leads recorded as described in previous studies [19]. UHF-ECG recordings were acquired for 1–10 minutes in a supine position during the pacemaker implantation procedure.

During the implantation procedure, patients underwent temporary application of various conventional and physiological pacing techniques, after which the final pacing strategy was selected at the discretion of the implanting physicians. Conventional pacing included RVAP and RVSP. Physiological pacing included various subtypes of HBP and LBBAP. HBP subtypes were selective HBP (sHBP), characterized by pure His capture pacing, and nonselective HBP (nsHBP), which involved the capture of both the His bundle and adjacent myocardial tissue. The LBBAP subtypes were: selective LBB pacing (sLBBP), involving exclusive LBB capture; nonselective LBB pacing (nsLBBP), with capture of both the LBB and the adjacent left septal myocardium; and LV septal pacing (LVSP), when the lead failed to capture the LBB but reached the LV subendocardium. In some patients, multiple recordings were collected in spontaneous rhythm and/or under some pacing types. In those cases, the first available recording was used for paired analysis.

The distribution of ECG recordings in spontaneous rhythm and different pacing types throughout the dataset was as follows. 37 recordings (from 37 patients) were recorded for RVAP, 102 (from 87 patients) for RVSP, 50 (from 47 patients) for sHBP, 160 (from 137 patients) for nsHBP, 13 (from 11 patients) for sLBBP, 47 (from 40 patients) for nsLBBP, and 87 (from 50 patients) for LVSP. In addition, 199 recordings (from 170 patients) were acquired during spontaneous heart rhythm. All patients signed an informed consent prior to enrollment.

### 2.2 Pacemaker implantation

For HBP, the procedure described in previous work was followed [8]. The His bundle region was mapped using a SelectSecureTM lead (model 3830, 69 cm, Medtronic Inc., Minneapolis, MN), delivered through a fixed-curve sheath (C315HIS, Medtronic). sHBP and nsHBP captures were defined as previously described [12,23]. For LBBAP, the lead was moved toward the right ventricle (RV) along a line between the His bundle region and the RV apex and was screwed deep into the septum to obtain a position on the left side of the interventricular septum showing a paced QRS morphology of RBBB/pseudo-RBBB in lead V1 [24]. sLBBP, nsLBBP, and LVSP were described as in previous studies [24,25]: In brief, concomitant myocardial and LBB capture (non-selective LBBP) was defined by a pseudo-RBBB morphology with the terminal r/R in V1 during pacing with an output of 5 V at 0.5 ms, which changed to sLBBP or LVSP when decreasing the pacing output. Selective capture of the LBB (sLBBP) was observed after decreasing the pacing output from nsLBBP, with an unchanged R wave peak time in V5. Pure myocardial capture of the left septum without LBB capture (LVSP) was characterized by a prolonged R wave peak time in V5 (> 10 ms) after decreasing the pacing output from nsLBBP. [24]. For RVAP and RVSP, the right ventricular leads were implanted in a standard form at the apex or septum of the RV [12,23].

### 2.3 UHF-ECG data analysis

#### 2.3.1 Preprocessing.

ECG signals were high-pass filtered to remove baseline wander. A 50 Hz notch digital filter was applied to attenuate power line interference.

#### 2.3.2 Pacing spike removal.

**Fig 1:**
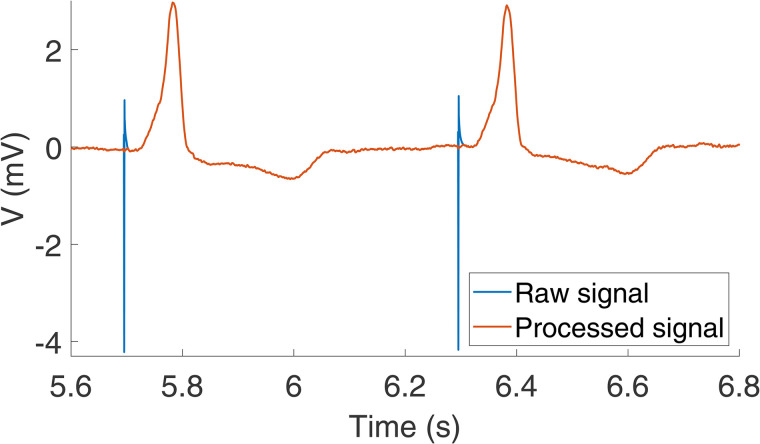
Pacing spike cancellation in a segment of an ECG lead. The signals before and after removing the pacing spike are shown in blue and red, respectively.

A semi-automated algorithm was developed to remove pacing artifacts, which departed from the method described in [32]. The algorithm was as follows:

1. Orthogonal leads XYZ were obtained from the standard 12-lead ECG using the Kors transformation matrix [33]. The vector magnitude, *v*(*n*), was computed as:
  $$v(n)=||v(n)||=\sqrt{x^{2}(n)+y^{2}(n)+z^{2}(n)}$$ (1)
  where ∣∣v(n)∣∣ is the vector norm of v(n), with *x*(*n*), *y*(*n*), and *z*(*n*) being the three orthogonal leads in v(n).
2. The magnitude slope, *dv*/*dt*, was calculated from the difference between consecutive samples of the time series *v*(*n*). The onset and end of the pacing artifact were selected as the first sample *n*_*o*_ and the last sample *n*_*e*_ for each beat of the time series *v*(*n*), respectively, that satisfied the following conditions:
  $$\begin{array}{c} \frac{dv(n)}{dt}>\alpha_{on}\\ \frac{dv(n)}{dt}<\alpha_{end} \end{array}$$ (2)
  where $\alpha_{on}$ and $\alpha_{end}$ are thresholds to determine the onset and end of the pacing spike. In this study, the threshold values were set to $\alpha_{on}=0.5$ mV/ms and $\alpha_{end}=-0.5$ mV/ms.
3. The pacing spike was removed by linear interpolation between the signal amplitude at *n*_*o*_ and *n*_*e*_. Fig 1 shows a segment of an ECG lead before and after removing the pacing spike by applying the described algorithm.

#### 2.3.3 Median beat calculation.

The QRS complexes in each signal were detected and classified according to their morphology, following previously proposed methods [34]. Subsequently, the delineation of the peaks, onsets, and ends of all ECG waves was performed using a wavelet-based single-lead automatic delineation software [35].

A representative beat for each ECG recording was defined as the median beat of all beats presenting the dominant morphology. The calculation of the median beat included the following steps:

1. RR intervals were computed from the fiducial points of the QRS complex.
2. The statistical mode of the RR interval time series was calculated for each recording. An RR histogram was computed using 20-ms bins. The bin containing the RR mode was selected, and the median of the beats corresponding to the RR intervals in that bin was computed.
3. All beats within the RR bin were aligned with respect to the computed median beat by maximizing the cross-correlation.
4. The rank correlation between the initially calculated median beat and each of the aligned beats was calculated. Beats with rank correlation coefficients below 0.85 were discarded.
5. The final median beat was computed from the aligned beats that were not discarded.

#### 2.3.4 Depolarization indices.

The single-lead local depolarization duration (Vxd) and electrical dyssynchrony in the ventricles (e-DYS) were calculated from UHF-ECG by first dividing the spectrum from 150 Hz to 1,000 Hz into 16 frequency bands, following the method described in [19]. For each band and precordial lead (V1-V8), the amplitude envelopes were calculated using the Hilbert transform, with the amplitude envelopes segmented according to the QRS onset and end annotations. The median amplitude envelope in each frequency band was normalized so that its integral was one. e-DYS was calculated as the maximal time difference between the center of mass of the QRS above the 50 percent threshold of the baseline-to-peak amplitude for the leads V1-V8. The sign of e-DYS was associated with the order of ventricular activation. Negative e-DYS values indicated that the LV was activated earlier than the right ventricle.

Additionally, the local activation duration in a given lead, defined between the crossings at half the maximum peak magnitude of the average of the normalized median envelopes in that lead, was defined as Vxd. Low Vxd values were related to high apparent conduction velocities, presumably due to the contribution of the intrinsic conduction system, while high Vxd values were related to a nonhomogeneous substrate or nonphysiological origin of myocardial propagation. Vd was calculated as the mean of all the computed Vxd values and represented the average duration of depolarization propagation.

The duration of the QRS (QRS_d_) was measured in each median beat (computed as described in section 2.3.3) as the time between the onset and end of the QRS complex, with QRS onset and end marks obtained from the wavelet-based delineator in each lead and the subsequent application of post-processing rules to obtain a unique multilead QRS_d_. In particular, QRS onset was identified as the earliest delineation mark of the individual leads that had at least 3 neighboring marks within an interval of 12 ms, and QRS end was selected as the latest delineation mark of the individual leads whose 3 nearest neighbors were within an interval of 10 ms [35,36]. The same measurement approach was applied to both paced and intrinsic QRS complexes.

**Fig 2:**
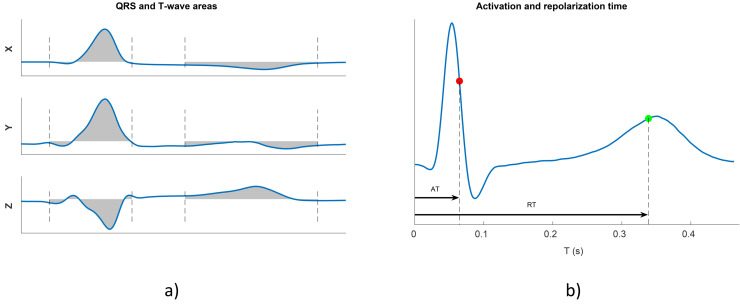
a) QRS and T-wave areas (shaded areas) in orthogonal leads. b) ECG beat for one lead with identified time points used to compute AT (red) and RT (green), calculated from the multilead QRS onset.

From the 8 independent leads of the median beat, the vectorcardiogram (VCG) was synthesized using the Kors method [37]. The QRS area (QRS_a_) in each orthogonal lead (X, Y, and Z) was calculated as the integral of the absolute value of the QRS complex from the beginning to the end of the QRS, as illustrated in Fig 2a). The total QRS_a_ was calculated as:


$$QRS_{a}=\sqrt{{QRS}_{a,X}^{2}+{QRS}_{a,Y}^{2}+{QRS}_{a,Z}^{2}}$$ (3)

where QRS_a,X_, QRS_a,Y_, and QRS_a,Z_ denote the QRS areas in leads X, Y, and Z.

In addition, the local activation time (AT) was calculated for each individual precordial lead as the center of mass of the absolute QRS complex of the representative median beat, using the multilead QRS onset as a reference. An example is shown in Fig 2b), where the AT is marked with a red point. The difference between the last and the first AT values within the 6 precordial leads V1-V6 was defined as the dispersion of the activation times. This dispersion was calculated as the absolute value of the difference (denoted dAT) or by including a negative sign when the number of the first activated lead (1–6 for V1 to V6) was greater than that of the last activated lead (denoted dATs).

To specifically assess the activation dyssynchrony within the LV, analogous measurements of AT dispersion were calculated focusing only on leads V4-V6. These variables were denoted as dAT_4-6_ and dATs_4-6_ when the absolute value and the signed value of AT dispersion in V4-V6 were measured, respectively.

The results in the following section are presented using ΔX for each calculated marker X. ΔX denotes the difference between the value of marker X under a given pacing modality and the corresponding value in spontaneous rhythm.

#### 2.3.5 Repolarization indices.

The QT interval, which represents the time needed for ventricular activation and repolarization, was calculated as the interval between the multilead QRS complex onset and the multilead T-wave end of the median beat, determined by applying post-processing selection rules to the delineation marks of individual leads [35]. Taking into account the QT intervals of all recordings in the dataset, a correction for the effects of heart rate was performed. To derive the correction formula, different regression models were first fitted to the QT and RR measurements, with the regression models being linear, hyperbolic, parabolic, logarithmic, shifted logarithmic, exponential, arc tangent, hyperbolic tangent, arc hyperbolic sine, and arc hyperbolic cosine [38]. The regression model leading to the lowest residual from the fitting was selected, and the corresponding correction formula was derived by projecting the QT interval to a standard level of RR equal to one second. The corrected QT intervals calculated according to the derived formula were denoted by QTc. This formula was QTc = QT + 0.196· (1-RR), where QT and RR intervals are measured in seconds.

In each precordial lead, the repolarization time (RT) was determined as the center of mass of the absolute T wave of the representative median beat (represented as a green point in Fig 2b), measured from the multilead QRS onset mark. A corrected RT, denoted as RTc, was calculated by deriving a correction formula analogous to the QT interval correction to remove any RR influence on RT. The correction formula in this case was RTc = RT + 0.123· (1-RR), where RT and RR intervals are measured in seconds. The dispersion of RT, denoted as dRT, was calculated as the difference between the maximum and minimum RTc values in the 6 precordial leads V1-V6. The signed dispersion of RT, denoted as dRTcs, included a negative sign to the value of dRTc when the number of the first repolarized lead (1–6 for V1 to V6) was higher than that of the last repolarized lead.

In a similar manner to dAT_4-6_ calculation, RT dispersion within the LV was specifically calculated by focusing the analysis on the leads V4-V6 only. The corresponding variables were denoted as dRTc_4-6_ and dRTcs_4-6_.

Based on the AT and RT variables, the activation–recovery interval (ARI) was calculated for each recording from the difference between the corresponding RT and AT values. Subsequently, a heart-rate-corrected ARI measurement (ARIc) was calculated using an approach similar to that used to compute QTc and RTc.

The T-wave area (T_a_) was calculated from the orthogonal leads X, Y, and Z according to the following formula:


$$T_{a}=\sqrt{T_{a,X}^{2}+T_{a,Y}^{2}+T_{a,Z}^{2}}$$ (4)

where T_a,X_, T_a,Y_, and T_a,Z_ denote the T-wave areas in leads X, Y, and Z. An example of the calculation of the T-wave area is shown in Fig 2a).

**Fig 3:**
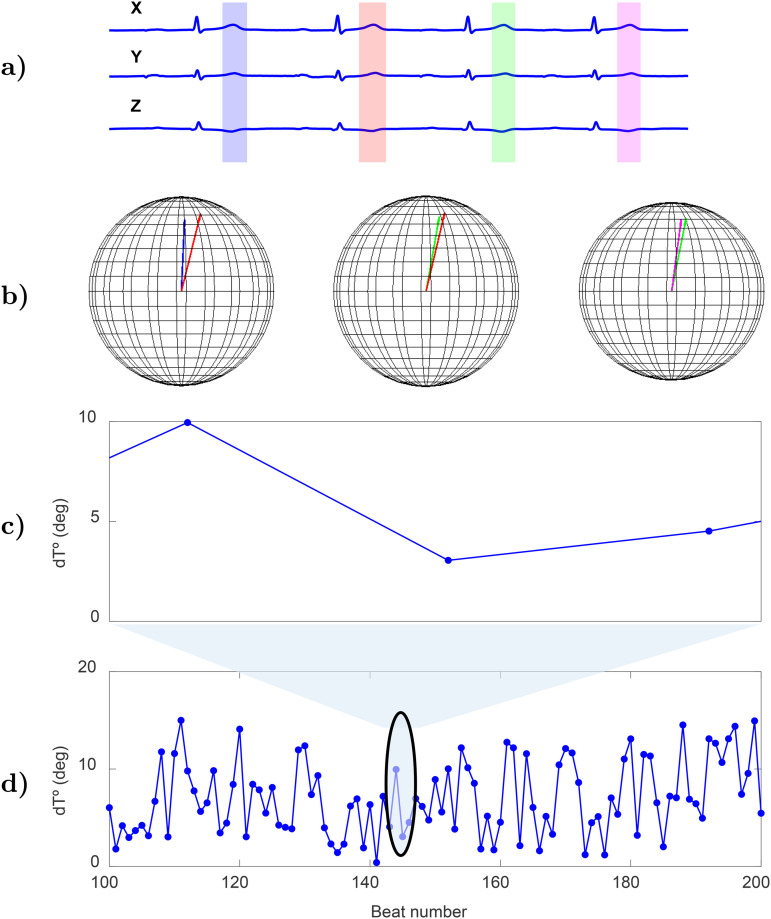
Steps for PRD calculation from ECG recordings in the Frank lead configuration. a) T waves for four consecutive beats. b) Three-dimensional representation of each pair of T-wave vectors. c) Angle between two consecutive T-wave vectors, dT°, for the represented beats. d) dT° time series along 100 consecutive beats.

Another studied repolarization marker was the Periodic Repolarization Dynamics (PRD), which was proposed to assess sympathetic modulation of ventricular repolarization by measuring low-frequency (below 0.1 Hz) oscillations in the T-wave vector. The PRD index was computed using the method described in [39], which represents a modified version of the method originally published in [40]. The method included the following steps:

1. The T wave of each cardiac beat was segmented by defining a window dependent on the QRS fiducial point and the RR interval corresponding to that beat. The onset of the T-wave window, denoted by $T_{on_{i}}$, was set at 90 ms after the fiducial mark QRS_i_: $T_{on_{i}}=QRS_{i}+90\;ms$. The end of the T-wave window, denoted by $T_{end_{i}}$, was defined as $T_{end_{i}}=QRS_{i}+min(360\;ms,\frac{2}{3}RR_{i})$ for RR_i_ below 720 ms and $T_{end_{i}}=QRS_{i}+360\;ms$ in other cases.
2. A constant value was subtracted from each T wave in each of the analyzed leads so that the voltage at the end of the T-wave window was set to 0 mV.
3. The average electrical vector was calculated for each T-wave window. The angle dT° between two consecutive T-wave windows was calculated from the dot product of the normalized average vectors.
4. A 10th-order median filter was applied to attenuate outliers and artifacts in the dT° time series.
  Fig 3 illustrates steps (i) to (iv) and the resulting dT° time series over 100 beats.
  From the calculated dT° time series, a method based on phase rectified signal averaging (PRSA) [41] was applied to evaluate the magnitude of its low-frequency oscillations, which are measured by the PRD index [42]. This evaluation involved the following steps:
5. Anchor points were defined by comparing the averages of the *M* = 9 values of the series dT° before and after each candidate anchor point $x_{\text{i}}$. A point $x_{\text{i}}$ was considered to be an anchor point if:
  $$\frac{1}{M}\sum_{j=0}^{M-1}x_{i+j}>\frac{1}{M}\sum_{j=1}^{M}x_{i-j}$$ (5)
  The value of *M* = 9 was established because it allows for detection of frequencies in the range of interest (from 0.025 to 0.1 Hz), as fully described in [41].
6. Windows of 2*L* values were defined around each anchor point. Anchor points in the first and last *L* samples of the dT° series were discarded, as windows of length 2*L* could not be defined around them. In this study, *L* = 20 was chosen, as it allowed for the detection of frequencies in the range of interest.
7. The PRSA series was obtained by averaging the dT° values over all 2*L*-sample windows around anchor points contained in each recording.

PRD was defined as the difference between the maximum and minimum values of the PRSA series.

Analogous to the calculation of ΔX for depolarization markers, differences were calculated between the value of the marker X under a given pacing modality and the corresponding value in spontaneous rhythm.

### 2.4 Statistical analysis

Continuous and discrete variables are presented as median [interquartile range (IQR)] and counts (percentages), respectively. The Wilcoxon signed-rank test was used for paired comparison of continuous variables, such as those performed to assess the statistical significance of the differences between a marker measured under a pacing modality and the same marker measured in spontaneous rhythm. For unpaired comparisons, the Mann–Whitney U test (or Wilcoxon rank-sum test) was applied when evaluating differences in the Δ indices between a pacing modality and RVAP. All statistical analyses were performed with MATLAB R2020a (9.8). Differences were considered statistically significant if the associated p-value < 0.05. NS is used to denote nonsignificant.

## 3 Results

### 3.1 Depolarization indices

#### 3.1.1 QRS duration.

**Fig 4:**
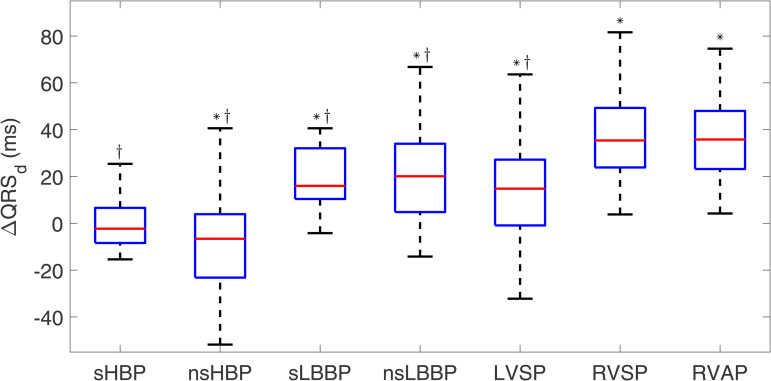
Box plots of $\Delta QRS_{d}$ for each of the pacing types with respect to spontaneous rhythm. ^*^p<0.05 with respect to spontaneous rhythm, ^†^*p*<0.05 with respect to RVAP.

Fig 4 shows the median and IQR of $\Delta QRS_{d}$ for all the pacing types analyzed in this study. QRS complexes in spontaneous rhythm had a median duration of 103.6 [18.4] ms. The widest QRS complexes were observed after RVP, both RVSP (median [IQR] of $\Delta QRS_{d}$ equal to 35.0 [25.5] ms, p < 0.001 compared to spontaneous rhythm) and RVAP (35.8 [24.8] ms, p < 0.001). LBBAP led to shorter QRS complexes than RVP, although still significantly longer than those for spontaneous rhythm (with increases of 16.0 [21.7] ms, 20.1 [29.2] ms and 14.8 [28.1] ms for sLBBP, nsLBBP, and LVSP, respectively, with respect to spontaneous rhythm, p < 0.001). sHBP stimulation induced median QRS_d_ values similar to those in spontaneous rhythm (with changes of −2.3 [15.0] ms, NS), while nsHBP pacing was associated with the lowest QRS_d_ values (changes of −6.6 [27.1] ms, p-value<0.001 compared to spontaneous rhythm). All physiological pacing techniques showed significant differences (p < 0.001) compared to RVAP.

#### 3.1.2 Electrical dyssynchrony and activation duration.

**Fig 5:**
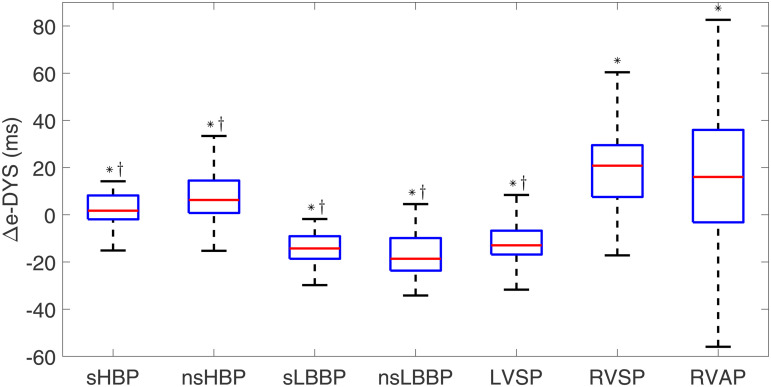
Box plots of Δ e-DYS for each of the pacing types with respect to spontaneous rhythm. ^*^p<0.05 with respect to spontaneous rhythm, ^†^*p*<0.05 with respect to RVAP.

**Fig 6:**
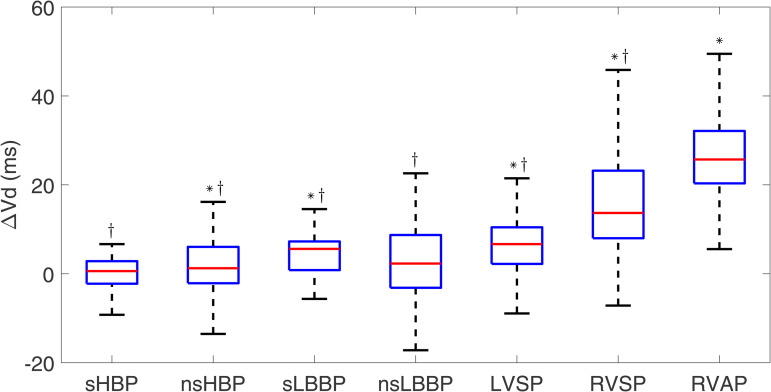
Box plots of Δ Vd for each of the pacing types with respect to spontaneous rhythm. ^*^p<0.05 with respect to spontaneous rhythm, ^†^*p*<0.05 with respect to RVAP.

Figs 5 and 6 show box plots for Δe-DYS and ΔVd. Similarly to the observations for $\Delta QRS_{d}$, RVP techniques (RVSP and RVAP) showed the largest dyssynchrony and activation duration (Δe-DYS: 20.8 [22.0] ms; ΔVd: 13.7 [15.2] ms for RVSP, and e-DYS: 16.0 [39.2] ms, Vd: 25.7 [11.8] ms for RVAP).

LBBAP techniques (sLBBP, nsLBBP, and LVSP) induced negative Δe-DYS values, indicative of higher ventricular synchrony than for spontaneous rhythm (−14.3 [9.6] ms, −18.6 [13.8] ms, and −12.9 [10.1] ms, p < 0.001 in the three cases). HBP techniques (sHBP and nsHBP) generated small positive Δe-DYS values (1.7 [10.1] ms and 6.3 [13.7] ms, respectively, p = 0.02 and p < 0.001). Both HBP and LBBAP techniques were associated with small ΔVd values, indicating that Vd was similar to that of spontaneous rhythm.

Statistically significant differences were found for all physiological techniques with respect to RVAP, in terms of both Δe-DYS and ΔVd.

#### 3.1.3 QRS area.

**Fig 7:**
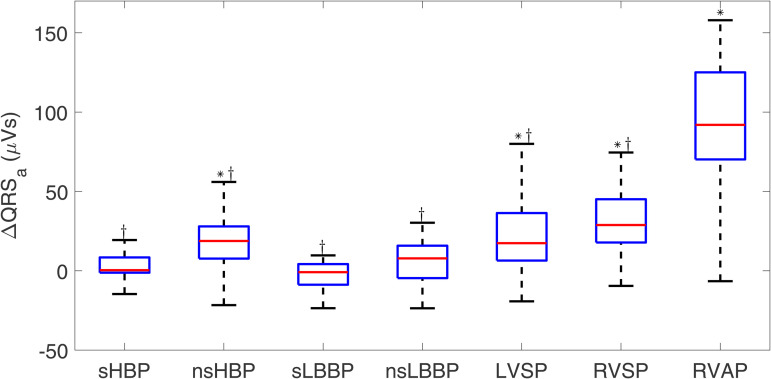
Box plots of $\Delta QRS_{a}$ for each of the pacing types with respect to spontaneous rhythm. ^*^p<0.05 with respect to spontaneous rhythm, ^†^*p*<0.05 with respect to RVAP.

The values of QRS_a_ during spontaneous rhythm were 50.06 [27.23] μVs. The highest differences in QRS_a_ with respect to spontaneous rhythm were found for RVAP and RVSP, which presented increases of 28.8 [27.3] μVs and 92.0 [54.9] μVs (p-values < 0.001 with respect to spontaneous rhythm). sLBBP, nsLBBP, and sHBP showed the lowest $\Delta QRS_{a}$ values, which were close to zero (−0.9 [13.0] μVs, 7.8 [20.4] μVs, and 0.4 [9.6] μVs) Box plots for $\Delta QRS_{a}$ are found in Fig 7.

All physiological techniques yielded significantly lower values of $\Delta QRS_{a}$ than RVAP.

#### 3.1.4 Activation time patterns.

**Fig 8:**
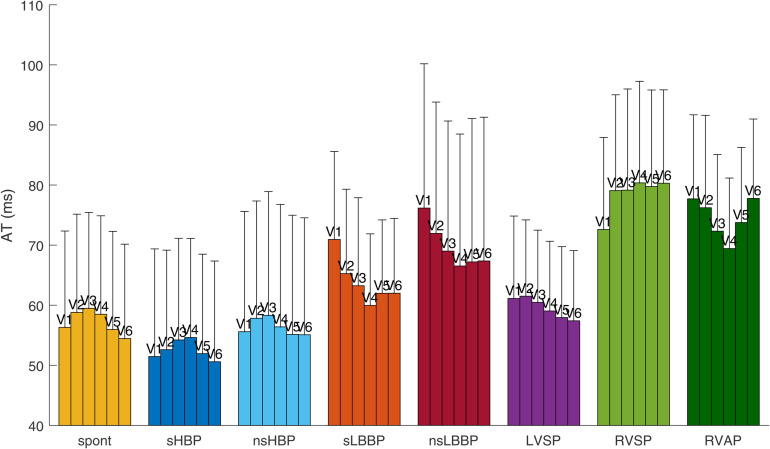
Average activation time (AT) in precordial leads V1-V6.

Fig 8 shows the average AT values in the precordial leads V1-V6 for spontaneous rhythm and each cardiac pacing type. As can be seen in the figure, leads V1, V5, and V6 present the earliest activation in spontaneous rhythm (54.5 ms in lead V6), while V2-V4 present the latest activation (59.5 ms in lead V3). Similarly, sHBP and nsHBP show activation patterns in which the regions corresponding to leads V1, V5, and V6 are activated earlier than those corresponding to leads V2-V4. LBBAP pacing types, as expected, present the earliest activations in leads V4-V6 (earliest activation at 60.0 ms for sLBBP, 66.5 ms for nsLBBP, and 57.4 ms for LVSP), while the latest activations are found in leads V1-V3 (latest activation at 70.9 ms, 76.2 ms, and 61.5 ms, respectively). The AT values for RVP types are the largest in practically all leads compared to other pacing types and spontaneous rhythm. For RVAP, the activation starts in V4 and ends in V1 and V6. For RVSP, the activation starts in V1 and ends in V4 and V6.

#### 3.1.5 Dispersion of activation time.

**Fig 9:**
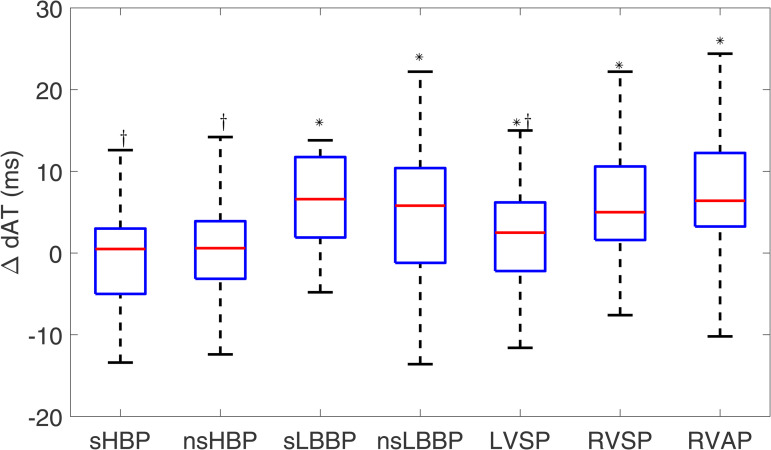
Box plots of ΔdAT for each of the pacing types with respect to spontaneous rhythm. ^*^p<0.05 with respect to spontaneous rhythm, ^†^*p*<0.05 with respect to RVAP.

The median dAT for spontaneous rhythm was 8.80 [5.00] ms. As shown in Fig 9, sHBP and nsHBP rendered the closest dAT values to the intrinsic rhythm (ΔdAT: 0.5 [8.0] ms and 0.6 [7.1] ms, respectively, NS for both cases). LBBAP and RVP techniques presented significantly higher dAT values, with ΔdAT: 6.6 [9.9] ms, 5.8 [11.6] ms, 2.5 [8.4] ms, 5.0 [9.0] ms, and 6.4 [9.0] ms for sLBBP, nsLBBP, LVSP, RVSP, and RVAP, respectively, p < 0.001 with respect to the intrinsic rhythm.

When considering the sign of dispersion, sHBP and nsHBP exhibited median values of ΔdATs close to zero (0.0 [17.6] ms and 0.6 [23.4] ms, respectively), indicating high similarity to spontaneous rhythm. sLBBP, nsLBBP, and LVSP rendered slightly negative ΔdATs values, representative of lower dATs (−6.8 [13.5] ms, −7.0 [21.1] ms, and −2.8 [22.8] ms, p < 0.05, respectively). RVSP was associated with the highest ΔdATs values (8.2 [26.4] ms, p < 0.05). The corresponding box plots are presented in S1 Fig.

**Fig 10:**
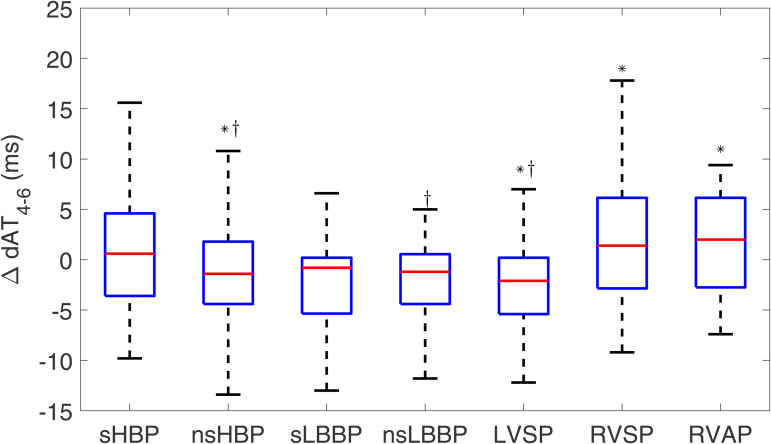
Box plots of $\Delta {dAT}_{4-6}$ for each of the pacing types with respect to spontaneous rhythm. ^*^p<0.05 with respect to spontaneous rhythm, ^†^*p*<0.05 with respect to RVAP.

The results for the dispersion of activation times within the LV (dAT_4-6_) are shown in Fig 10. Both sHBP and nsHBP led to lower $\Delta {dAT}_{4-6}$, which was not significantly different from zero (0.6 [8.2] ms and −1.4 [6.2] ms, respectively). sLBBP and nsLBBP also had median values close to zero (−0.8 [5.6] ms and −1.2 [5.0] ms, NS). Conventional pacing types had the highest values (1.4 [9.0] ms for RVSP and 2.0 [8.9] ms for RVAP, p < 0.05 in both cases). The corresponding results for $\Delta {dATs}_{4-6}$ are presented in S2 Fig.

For ΔdAT, sHBP, nsHBP, and LVSP led to values significantly lower than RVAP. For $\Delta {dAT}_{4-6}$, significantly lower values than those for RVAP were found for nsHBP, nsLBBP, and LVSP.

### 3.2 Repolarization indices

#### 3.2.1 Corrected QT.

**Fig 11:**
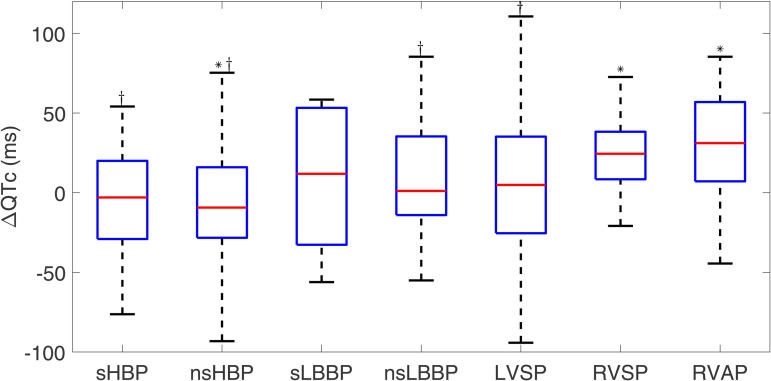
Box plots of ΔQTc for each of the pacing types with respect to spontaneous rhythm. ^*^p<0.05 with respect to spontaneous rhythm, ^†^*p*<0.05 with respect to RVAP.

The median QTc under spontaneous rhythm was 429 [43] ms. As shown in Fig 11, sHBP and nsHBP led to ΔQTc values lower than zero (−3.0 [49.0] ms, NS, and −9.4 [44.3] ms, p < 0.05, respectively). Of the LBBAP techniques, nsLBBP led to small positive ΔQTc (1.1 [49.4] ms, NS), while sLBBP led to slightly higher values (11.8 [85.9] ms, NS). RVSP and RVAP induced larger QTc prolongations (ΔQTc: 24.4 [29.8] ms and 31.1 [49.8] ms, p < 0.05). QTc was significantly shorter under sHBP, nsHBP, nsLBBP, and LVSP than under RVAP.

#### 3.2.2 T-wave area.

**Fig 12:**
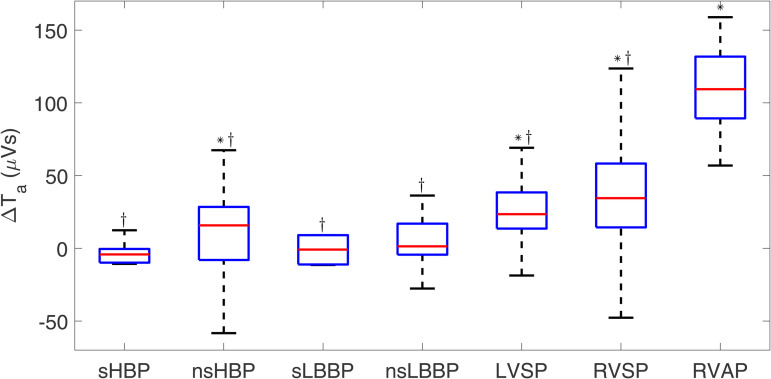
Box plots of $\Delta T_{a}$ for each of the pacing types with respect to spontaneous rhythm. ^*^p<0.05 with respect to spontaneous rhythm, ^†^*p*<0.05 with respect to RVAP.

Fig 12 shows the values of $\Delta T_{a}$ for each type of cardiac pacing. Interestingly, the observed trend is very similar to that observed for $\Delta QRS_{a}$, shown in Fig 7. The value of T_a_ for spontaneous rhythm was 30.50 [27.83] μVs. The highest $\Delta T_{a}$ values were found for RVSP (34.5 [43.9] μVs, p < 0.001) and RVAP (109.4 [42.5] μVs, p < 0.001). Physiological pacing approaches, particularly sHBP, sLBBP, and nsLBBP, presented $\Delta T_{a}$ values close to zero, which means similar T-wave areas to those found for spontaneous rhythm (sHBP: −4.2 [9.4] μVs, NS; sLBBP: −0.9 [20.1] μVs, NS; nsLBBP: 1.3 [21.3] μVs, p < 0.05). All physiological pacing techniques rendered smaller T-wave areas than RVAP.

#### 3.2.3 Periodic repolarization dynamics.

**Fig 13:**
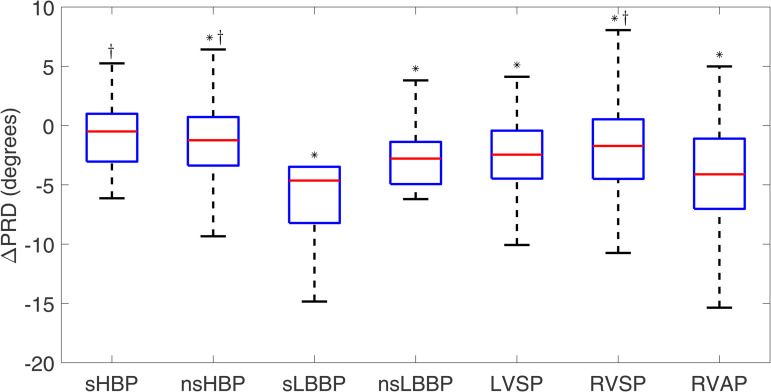
Box plots of Δ PRD for each of the pacing types with respect to spontaneous rhythm. ^*^p<0.05 with respect to spontaneous rhythm, ^†^*p*<0.05 with respect to RVAP.

For spontaneous rhythm, the PRD median value was 4.94 [3.93] degrees. As illustrated in Fig 13, ΔPRD took negative values for all cardiac pacing techniques. The lowest ΔPRD values were found for sLBBP and RVAP (−4.6 [4.7] and −4.1 [5.9] degrees, respectively). In the case of sHBP, its ΔPRD value was close to zero (−0.5 [4.0] degrees, NS). Of the evaluated physiological pacing modes, statistically significant differences with respect to RVAP were found for sHBP, nsHBP, and RVSP.

#### 3.2.4 Repolarization time patterns.

**Fig 14:**
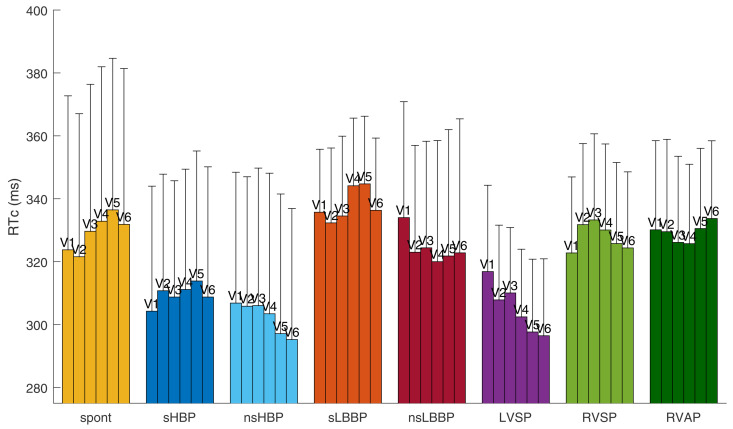
Average repolarization time (RT) corrected by RR duration in precordial leads V1-V6.

Fig 14 shows the average RT values corrected for the RR interval in the precordial leads V1-V6 for spontaneous rhythm and each cardiac pacing type. For spontaneous rhythm, the first lead to repolarize was V2 (321.6 ms), with lead V5 being the last to repolarize (336.5 ms). Similar RT patterns were found for the two types of selective physiological pacing, i.e., sHBP and sLBBP, with the earliest repolarization occurring in V1-V2 and the latest in V5. The RT patterns for nsHBP, nsLBBP, and LVSP were similar to the corresponding AT patterns, with V4, V5, and V6 being the first leads to repolarize in each case. RVAP showed RT patterns similar to those of AT; however, in this case, lead V6 was the last to repolarize. For RVSP, the repolarization took longer to complete in lead V3.

#### 3.2.5 Dispersion of repolarization time.

**Fig 15:**
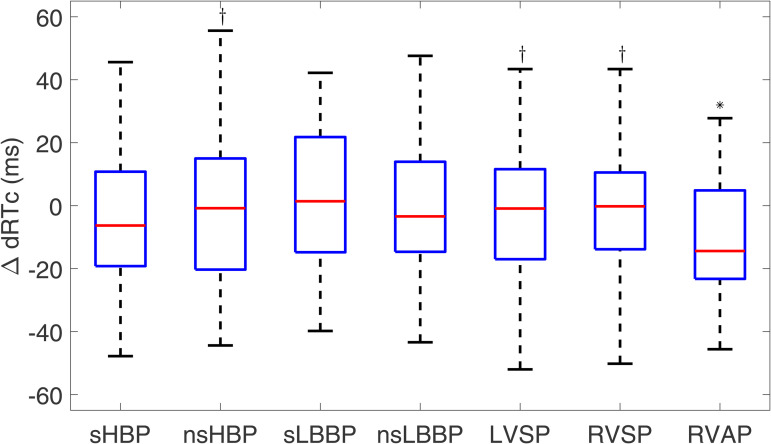
Box plots of ΔdRTc for each of the pacing types with respect to spontaneous rhythm. ^*^p<0.05 with respect to spontaneous rhythm, ^†^*p*<0.05 with respect to RVAP.

The median value of dRTc for spontaneous recordings was 31.2 [22.2] ms. Fig 15 shows that ΔdRTc was comparable for all pacing types, with the lowest values observed for RVAP (−14.4 [28.1] ms).

**Fig 16:**
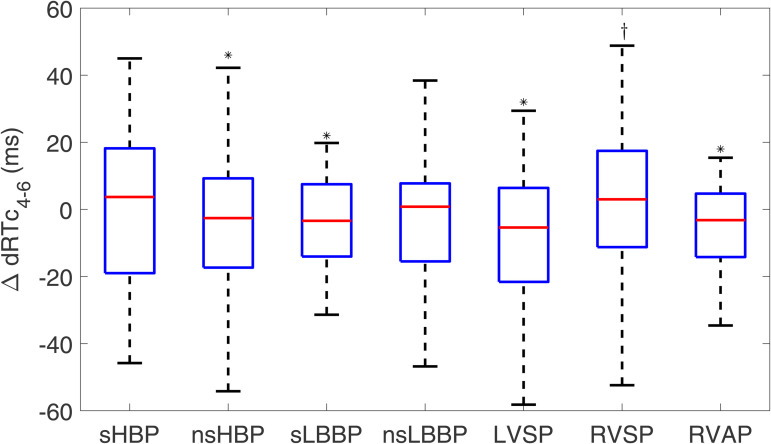
Box plots of $\Delta {\text{dRTc}}_{4-6}$ for each of the pacing types with respect to spontaneous rhythm. ^*^p<0.05 with respect to spontaneous rhythm, ^†^*p*<0.05 with respect to RVAP.

When conducting the analysis for $\Delta {\text{dRTc}}_{4-6}$, all pacing techniques showed small values, with the lowest median value found for LVSP (−5.4 [28] ms), as shown in Fig 16.

The corresponding results for ΔdRTcs and $\Delta {\text{dRTcs}}_{4-6}$ are presented in S3 and S4 Figs.

In addition to activation and repolarization times, the results for ΔARIc are presented in S5 Fig.

A disaggregated analysis for patients with atrioventricular block and those with sick sinus syndrome was performed to assess the response to each pacing technique in these two subpopulations. Despite minor quantitative differences, the results were qualitatively consistent across both groups.

## 4 Discussion

This study provides a detailed characterization of the response of ventricular depolarization and repolarization to seven different types of cardiac pacing in patients without abnormalities in ventricular conduction and with an indication for pacemaker implantation due to bradycardia. In addition to quantifying depolarization and repolarization indices commonly used in other studies, we propose additional markers to describe the spatial dispersion of the activation and repolarization times from UHF-ECG recordings. Properties related to the duration, area, and spatial heterogeneity of ECG activation and repolarization waves are shown to differentiate the effects of each of the tested physiological and conventional cardiac pacing techniques, with implications for the development of deleterious effects on cardiac function.

### 4.1 Pacing-induced effects on ventricular depolarization

For the characterization of cardiac pacing effects on ventricular depolarization, this study first examined classical ECG markers reported in the literature, such as the duration and area of the QRS complex. Other more recently proposed markers quantified from UHF-ECG recordings were also analyzed, including Vd (measuring local activation duration) and e-DYS (measuring activation dyssynchrony). Finally, novel markers that can be quantified from standard ECGs (not necessarily sampled at very high frequency) were proposed based on ATs in different ECG leads and their spatial dispersion across the entire ventricles and across the LV only.

From the analysis of all the described markers, we found that the physiological pacing techniques sHBP and nsHBP exhibit an electrical response comparable to that observed in the spontaneous rhythm in patients without any conduction disorders. LBBAP techniques, including sLBBP, nsLBBP, and LVSP, despite presenting larger differences with respect to spontaneous rhythm than HBP-based techniques, are able to preserve the synchrony in the activation of the LV, with only a delayed activation in the RV. The conventional pacing techniques RVSP and RVAP show markedly different responses compared to those found in spontaneous rhythm, with high dispersion in the ventricular ATs. This behavior was also observed in previous studies [23–25].

QRS_d_ is a measure commonly used to assess ventricular synchrony. Changes in QRS_d_ have been associated with the occurrence of major adverse cardiovascular events [43,44]. Here, we showed that sHBP and nsHBP had $\Delta QRS_{d}$ values close to zero, indicating similarities to spontaneous rhythm. The longest QRS_d_ values were found for RVSP and RVAP. The three LBBAP techniques, i.e., sLBBP, nsLBBP, and LVSP, were associated with similar values of $\Delta QRS_{d}$, which were higher than those for HBP. Our results are in line with those reported in previous studies that compared the ventricular activation synchrony of sLBBP and nsLBBP with that of LVSP [45] and RVAP [46]. The larger QRS_d_ values found for sLBBP, nsLBBP, and LVSP compared to spontaneous or His-paced activations can be attributed to the fact that the electrical pacing impulse is rapidly transmitted to the LV through the LBB or the myocardium close to it, while the RV is activated with some delay.

Activation indices derived from the VCG have been suggested to predict the response to cardiac resynchronization therapy more accurately than QRS_d_ [47]. Here, QRS_a_ was measured from the VCG. QRS_a_ has been reported to be less sensitive to uncertainty in the determination of the QRS boundaries, as the extremes of the QRS complex scarcely contribute to the area [48,49], and to present less variability in its measurement than QRS_d_ [50]. Previous works have reported QRS_a_ to be an index of dyssynchrony in electrical activation, with a large QRS_a_ corresponding to the delayed activation of the posterolateral wall of the LV, independently of QRS morphology [51]. Our results indicate that sHBP and sLBBP have QRS_a_ values similar to those in spontaneous rhythm, whereas nsHBP, nsLBBP, and LVSP have somewhat higher values that are still far from the large values associated with RVAP. Considering that a decrease in QRS_a_ has been shown to be an independent predictor of survival and reverse cardiac remodeling [52], the results of the present study indicate that physiological pacing, especially through selective HBP or LBBP techniques, entails significantly lower risk than conventional pacing techniques.

Using markers specifically derived from UHF-ECG recordings, such as e-DYS and Vd, the ventricular response to different pacing techniques has been investigated in previous studies [19,21,24]. These studies have shown that HBP produces physiological activation times and patterns, preserving fast activation of the LV lateral wall and avoiding any deterioration in interventricular synchrony. No significant differences between sHBP and nsHBP in terms of ventricular activation patterns were reported when tested in patients without conduction disorders. Our results are in line with those previous findings. The results for LBBAP indicated a reduction in e-DYS values for LBBAP with respect to intrinsic rhythm, in association with lower activation times in leads V4-V6. The e-DYS values for HBP were very close to those of intrinsic rhythm, in agreement with previously reported results [53–55].

Furthermore, we measured the local ATs from the precordial leads V1-V6 and quantified their spatial differences with the aim of characterizing the sequence of activation and its spatial dispersion [56]. Our results indicate that HBP techniques preserve the activation synchrony of spontaneous rhythm, as manifested by similar dAT values. For LBBAP techniques, the synchrony is reduced, but only because the RV activates later, whereas the synchrony in LV activation, quantified by dAT_4-6_, is higher than that for spontaneous rhythm. Conventional pacing through RVSP or RVAP leads to increased dispersion in the activation of the two ventricles or the LV alone. Increases in the dispersion of both ventricular activation and repolarization have been shown to be important in the genesis and maintenance of ventricular arrhythmias and sudden cardiac death [57,58].

### 4.2 Pacing-induced effects on ventricular repolarization

Differences in ventricular repolarization as a function of pacing techniques were investigated by measuring the QTc interval, the T-wave area, the PRD index reflecting the magnitude of the low-frequency oscillations in the T wave, and the RTs across the precordial leads V1-V6, together with their dispersion.

The QTc interval has been widely used in the literature to provide an overall measure of the duration of repolarization after compensating for the effects of heart rate. In previous works, patients with significantly prolonged QTc intervals who underwent permanent pacemaker implantation have been reported to face a higher risk of new-onset LV systolic dysfunction, cardiac death, ventricular arrhythmias, and sudden cardiac death [59–61]. Other studies have shown that the pacing-induced increase in QTc, with respect to intrinsic rhythm, was less pronounced for LBBP techniques than for RVSP [22]. A study including 55 patients with LBBB and an indication for cardiac resynchronization therapy (CRT) [62] showed that HBP did not lead to immediate significant changes in corrected QT and Tpeak–Tend intervals with respect to baseline. Our results are in line with such findings. In particular, we show that HBP and LBBAP (sLBBP, nsLBBP, LVSP) are associated with similar QTc values to spontaneous rhythm (represented by values of ΔQTc close to zero), or even lower values in the case of nsHBP, whereas RVSP and RVAP present significantly higher ΔQTc values, indicative of QTc prolongation.

T_a_ has been postulated to carry long-term prognostic information on cardiovascular mortality in apparently healthy populations [63–65]. Furthermore, T_a_ has been suggested to be a sensitive feature that characterizes the ventricular repolarization response to pacing, with the capacity to track minor ST-segment and T-wave abnormalities [65] and to serve as a marker that can predict the response to CRT [48,66]. The results of our work indicate that conventional pacing techniques, especially RVAP, lead to high values of T_a_, while physiological pacing techniques lead to T_a_ values similar to those measured under intrinsic rhythm. These findings extend those of previous work by adding the characterization of T_a_ in response to a large number of conventional and physiological pacing techniques [67–69] and confirm the suitability of HBP and LBBAP techniques in terms of their effects on ventricular repolarization.

PRD is an arrhythmic risk marker that measures the magnitude of low-frequency oscillations in the T-wave vector modulated by sympathetic nervous system activity. Increased PRD values have been shown to be a strong predictor of all-cause mortality, cardiac mortality, and ventricular arrhythmias in various cardiac diseases and conditions [39,40,70–74]. Our results show that all cardiac pacing techniques induce a decrease in the median PRD values found in spontaneous rhythm, with the decrease reaching approximately 5 degrees, in median, for sLBBP and RVAP. The basis for such results deserves further investigation. Although no significant correlation was found between PRD and heart rate, in accordance with previous reports [75], additional studies are warranted to fully rule out the impact of heart rate on pacing-induced reduction in PRD.

To further characterize the response of ventricular repolarization to cardiac pacing, we evaluated the dispersion of RTs across the six precordial leads V1-V6 and focused only on V4-V6. No significant differences are present between most cardiac pacing techniques and spontaneous rhythm. In general, the median values of ΔdRTc and $\Delta {\text{dRTc}}_{4-6}$ for all pacing modalities, except for RVAP, are close to zero. Even if enhanced dispersion of ventricular repolarization has been suggested as indicative of reverse remodeling [76,77] and a greater susceptibility to arrhythmias such as Torsades de Pointes [78], our results support the suitability of all physiological pacing techniques, including LBBAP techniques, based on their ability to reproduce the basal dispersion of LV repolarization. Such suitability is also supported by the findings achieved for ΔARIc.

In summary, our results confirm previous evidence of the large similarities between HBP, particularly sHBP, and spontaneous rhythm in patients without ventricular conduction disorders [21]. The LBBAP techniques, despite differing from spontaneous rhythm in some global measurements of ventricular AT and RT dispersion, have been confirmed to lead to high synchrony in LV activation and repolarization. These results provide additional evidence of the validity of both HBP and LBBAP [79]. Conventional RVP techniques, however, are associated with the highest global and LV-specific dyssynchrony characteristics, which could have long-term adverse effects on ventricular function [80]. Our research thus supports the use of LBBAP modalities as physiological pacing techniques that overcome the shortcomings associated with conventional RVP.

## 5 Study limitations and future work

This work included ECGs of patients who underwent different types of cardiac pacing. However, there is a large variability in the number of patients subjected to each type of pacing. In particular, only 13 recordings were available for sLBBP, while more than 150 recordings were available for nsHBP or spontaneous rhythm. Another limitation is the lack of access to follow-up information. Long-term follow-up studies with a large representative number of patients for each type of pacing are required to corroborate the present results.

It is also important to note that most of the recordings were obtained by applying one stimulation technique after another. Although there was some waiting time between the application of a type of cardiac stimulation and the next one, the influence of the so-called cardiac memory phenomenon [81], particularly on ventricular repolarization, cannot be ruled out. Therefore, studies with longer time intervals between the application of different types of stimulation or the use of recordings without sequential pacing would be required to confirm the present findings.

Currently, the number of hospitals and medical centers where UHF-ECG signals are recorded is limited. Future studies should confirm the clinical value of the proposed markers dAT, dRTc, dAT_4-6_ and dRTc_4-6_, which can be measured from standard ECGs, as an adjunct or substitute for the marker e-DYS quantified from UHF-ECG signals.

## 6 Conclusions

This study analyzed a set of markers to characterize ventricular depolarization and repolarization in response to seven different types of cardiac stimulation. Physiological stimulation using both HBP and LBBAP produced functional behaviors that were close to those of the intrinsic rhythm in patients without cardiac disorders, whereas conventional RV stimulation differed markedly. The differences between HBP and LBBAP were minor for most markers, particularly those related to LV depolarization and repolarization. These findings support the use of LBBAP and HBP as alternative physiological pacing options in patients with narrow QRS complexes. Selection between these approaches may reasonably depend on operator experience, technical feasibility, and individual patient characteristics.

## Supporting information

S1 FigBox plots of ΔdATs for each of the pacing types respect to spontaneous rhythm.^*^p<0.05 respect to spontaneous rhythm, ^†^*p*<0.05 respect to RVAP.(EPS)

S2 FigBox plots of $\Delta {dATs}_{4-6}$ for each of the pacing types respect to spontaneous rhythm.^*^p<0.05 respect to spontaneous rhythm, ^†^*p*<0.05 respect to RVAP.(EPS)

S3 FigBox plots of ΔdRTs for each of the pacing types respect to spontaneous rhythm.^*^p<0.05 respect to spontaneous rhythm, ^†^*p*<0.05 respect to RVAP.(EPS)

S4 FigBox plots of $\Delta {\text{dRTs}}_{4-6}$ for each of the pacing types respect to spontaneous rhythm.^*^p<0.05 respect to spontaneous rhythm, ^†^*p*<0.05 respect to RVAP.(EPS)

S5 FigBox plots of $\Delta {\text{ARI}}_{c}$ for each of the pacing types respect to spontaneous rhythm.^*^p<0.05 respect to spontaneous rhythm, ^†^*p*<0.05 respect to RVAP.(EPS)
